# Suxamethonium-Induced Hyperkalemia: A Short Review of Causes and Recommendations for Clinical Applications

**DOI:** 10.1155/2021/6613118

**Published:** 2021-02-25

**Authors:** Henrik Lynge Hovgaard, Peter Juhl-Olsen

**Affiliations:** ^1^Department of Anaesthesiology and Intensive Care, Regionshospitalet Viborg, Heibergs Alle 5A, Viborg 8800, Denmark; ^2^Department of Internal Medicine, Randers Regional Hospital, Skovlyvej 15, Randers 8930, Denmark; ^3^Department of Anaesthesiology, Aarhus University Hospital, Palle Juul-Jensens Boulevard 99, Aarhus 8200, Denmark; ^4^Department of Clinical Medicine, Aarhus University, Palle Juul-Jensens Boulevard 82, Aarhus 8200, Denmark

## Abstract

After the introduction of suxamethonium in 1953, cases of cardiac arrest during induction of anesthesia were recorded. In the following years, hyperkalemia was identified as the cause, and the connection to acetylcholine receptor modulation as the underlying molecular mechanism was made. Activation of the acetylcholine receptor with suxamethonium, acetylcholine, or choline causes an efflux of potassium to the extracellular space. However, certain pathological conditions cause acetylcholine receptor proliferation and the emergence of immature receptors capable of a larger potassium efflux to the bloodstream. These pathologic conditions include upper and lower neuron injuries, major burns, trauma, immobility, muscle tumors, muscular dystrophy, and prolonged critical illness. The latter is more important and relevant than ever due to the increasing number of COVID-19 patients requiring prolonged respiratory support and consequent immobilization. Suxamethonium can be used safely in the vast majority of patients. Still, reports of lethal hyperkalemic responses to suxamethonium continue to emerge. This review serves as a reminder of the pathophysiology behind extensive potassium release. Proficiency in the use of suxamethonium includes identification of patients at risk, and selection of an alternative neuromuscular blocking agent is imperative.

## 1. Introduction

Neuromuscular blocking agents are widely used to facilitate endotracheal intubation [1]. Suxamethonium provides not only excellent conditions for airway management [2] but may also cause adverse events such as malignant hyperthermia, rhabdomyolysis, or hyperkalemia. These adverse events are potentially life threatening, and it is thus crucial to identify patients at risk, and, in these patients, choose an alternative neuromuscular blocking agent [3].

This short review will explain the causes for suxamethonium-induced hyperkalemia, identify patients at risk, and determine the duration of temporary risk factors.

## 2. Search Strategy

We searched the PubMed database to ensure an adequate and current description of hyperkalemia following suxamethonium administration using the following terms:

(((“Succinylcholine”[MeSH Terms] OR “Succinylcholine”[Text Word]) OR “suxamethonium”) AND ((“Potassium”[MeSH Terms] OR “Potassium”[Text Word]) OR ((“Hyperkalemia”[MeSH Terms] OR “hyperkalemia^*∗*^”[Text Word]) OR “hyperpotassemia^*∗*^”[Text Word] OR “hyperkalemic^*∗*^”[Text Word]))).

One author screened the abstracts and included all case reports, observational, and clinical studies on lethal hyperkalemia with a probable relation to suxamethonium administration. Reviews were scrutinized for additional literature, and a backwards snowballing strategy was employed.

Many compounds of modern anesthesia have changed profoundly over the last decades, and therefore, only articles published after the year 2000 were screened systematically.

The search revealed only few publications on certain major risk factors for suxamethonium-induced hyperkalemia (burns, rhabdomyosarcomas). Selected reports published before the year 2000 were thus also included.

## 3. Pharmacology of Suxamethonium

Suxamethonium is a neuromuscular blocking agent consisting of two synthetically joined molecules of acetylcholine (ACh). These ACh molecules can activate the ACh receptor, and if a sufficient number of ACh receptors are activated, the muscle cell is depolarized. Muscular contraction following depolarization with suxamethonium is similar to ACh-mediated physiologic muscle contractions. However, suxamethonium remains bound to the ACh receptor much longer than physiologic ACh, thus inhibiting further ACh receptor activation. ACh receptor activation ceases when suxamethonium is degraded by the plasma butyrylcholinesterase.

## 4. Physiology of Suxamethonium-Induced Hyperkalemia

The ACh receptor consists of five subunits arranged around a high conductance cation channel. Suxamethonium, Ach, and their common metabolite, choline, can bind to and activate the ACh receptor [4]. Activation causes an influx of sodium and calcium to the cytoplasm and an efflux of potassium to the extracellular space. Mature, innervated muscle cells express amounts exceeding 5 million ACh receptors in the neuromuscular junction [4].

**Figure 1:**
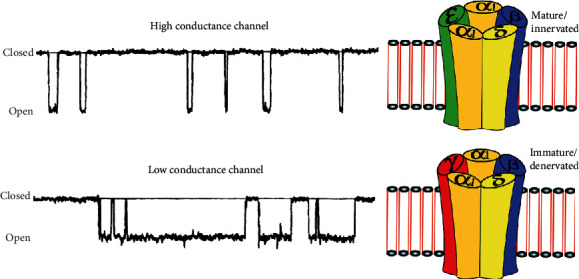
Outline of muscle acetylcholine receptor channels (right) and tracings of cell patch records of receptor channel openings (left). The acetylcholine receptor consists of five subunits. The mature receptor consists of two *α*1 subunits, and one each of *β*1, *δ*, and *ε* subunits. The immature isoform consists of two *α*1 subunits and one *β*1, *δ*, and *γ* subunit. The five subunits form a transmembrane cation channel. The immature receptor shows long opening times and low-amplitude currents and is therefore considered a low conductance channel. It may be depolarized with lower concentrations of agonists. The fact that these immature channels remain open for longer and are upregulated in certain pathologic states increases the chance that intracellular potassium ions will leak to the extracellular space following activation of the acetylcholine receptor with suxamethonium, acetylcholine, or choline and illustrated with permission from Wolters Kluwer [4].

Immature or denervated ACh receptors exhibit a slightly different subunit composition. Upon activation, these receptors stay open 2–10 times longer than the mature ACh receptor, thus facilitating a larger efflux of potassium down its concentration gradient (Figure 1) [4].

Pathologic conditions such as immobilization [5], denervation [6], and critical illness upregulate the ACh receptors by a factor of 2–100 not only in the neuromuscular junction but ACh receptors also proliferate along the muscle cell membrane [4]. The upregulated ACh receptors comprise more immature ACh receptors relative to the healthy neuromuscular junction. Therefore, in the mentioned pathologic conditions, the ACh receptors not only increase in numbers but also in distribution which together increases the transmembrane potassium conduction. The upregulation of ACh receptors produces a detectable rise in serum-potassium with suxamethonium administration, which is seen within six hours of, e.g., denervation. The rise in serum-potassium may reach a critical level as early as 72 hours postdenervation [4].

## 5. Clinical Implications

After the introduction of suxamethonium in 1951, cardiac arrests after induction of anesthesia were observed and first reported in burn patients [7]. The underlying mechanism, hyperkalemia, was not elucidated until 1967 [8], and shortly, thereafter, other pathologic conditions resulting in suxamethonium-induced hyperkalemia were identified [5]. In 1975, the probable underlying pathophysiology, ACh receptor upregulation as described above, was reviewed [9].

The following section comprises clinical conditions believed to cause ACh receptor modulation where the literature supports the potential for lethal hyperkalemia following suxamethonium administration.

### 5.1. Major Burns

In burn patients, the degree of ACh receptor proliferation corresponds to the magnitude of tissue damage [5]. Even modestly sized burn cases may be significant in this regard, and lethal hyperkalemia following suxamethonium administration has been observed in patients with a total burn surface of as little as 8% (equivalent to less than one arm) [10]. Lethal hyperkalemia in spite of only moderate tissue damage may be explained by systemic upregulation of the ACh receptor far from the injury site [11].

Reports from 1967 testify to patients tolerating suxamethonium in the first day postburn [10]. In current guidelines, suxamethonium administration in the first 48 hours postburn is considered safe [12]. Guidelines do not distinguish between chemical, electrical, and thermal burns.

In case of major burns, patients frequently undergo general anesthesia for debridement and other painful procedures. It was during such subacute procedures that the first cardiac arrests were recorded. In a 1967 case report during the Vietnam War, a young marine underwent 10 uneventful general anesthesias with suxamethonium in the first 26 days postburn. However, over the following weeks, he received five additional general anesthesias in which serum-potassium increased to >7 mmol/L. Three of these anesthesia episodes resulted in cardiac arrest; fortunately, the patient survived [13].

After attention was brought to the subject, suxamethonium was phased out in burn centers and no reports from the last two decades of neither hyperkalemia following induction of anesthesia nor toleration of suxamethonium in the years postburn were identified in the literature search for this review.

### 5.2. Critical Illness

Major activation of the inflammatory system, immobilization, and use of nondepolarizing neuromuscular blocking agents [14] are all possible features in critically ill patients believed to upregulate ACh receptors [6]. Numerous recent case reports testify to a lethal hyperkalemic response to suxamethonium administration in critically ill patients with a variety of somatic conditions [15–22] but also after bedrest in imminent preterm labor [23], bedrest without severe somatic disease [24], and catatonia [25].

In a 2012 study [26], the authors attempted to delineate the rise of serum-potassium following suxamethonium administration. In 156 intensive care unit (ICU) patients, serum-potassium was measured minutes before and after suxamethonium administration. The median rise in serum-potassium was 0.4 mmol/l (IQR: 0.0–0.7 mmol/l). The only independent risk factor for a rise in serum-potassium to more than 6.5 mmol/L was length of ICU stay exceeding 16 days. The authors found no significant correlation between rise in serum-potassium and patient neurologic deficits, prior to administration of nondepolarizing neuromuscular blocking agents or severe sepsis [27].

Critical illness and immobilization may produce prolonged muscle dysfunction for up to five years after hospital discharge [4]. The exact relationship between clinical loss of muscle strength, the expression of ACh receptors, and suxamethonium-induced hyperkalemia remains unclear.

### 5.3. Neurological Disease

Denervation is the most severe form of immobilization, and suxamethonium should be avoided in virtually all patients with suspected or confirmed denervation [28]. Both upper and lower motor neuron lesions facilitate ACh receptor proliferation along the muscle cell membrane well beyond the neuromuscular junction. This occurs as early as 48 h after the injury [4]. For this reason, suxamethonium is widely considered contraindicated in quadriplegia and paraplegia, but lethal hyperkalemia following suxamethonium administration has also recently been observed in patients with other conditions, undiagnosed at the time of suxamethonium administration, such as multiple sclerosis [29], amyotrophic lateral sclerosis [30], and Gordon syndrome [31].

### 5.4. Temporary Neurologic Disorder

Nonpermanent neurologic changes reported to cause lethal hyperkalemia include Guillain-Barré syndrome [32], botulism [33], and critical illness neuropathy as described above.

The upregulation of ACh receptors may persist as long as the neurological disorder is unresolved. The immature ACh receptor has a relatively short half-life (≈24 h) compared with the mature Ach receptor (≈1-2 weeks) [4]. Patients with temporary paralysis therefore theoretically recover to their normal levels of and relative distributions of ACh receptor types within a few weeks. Despite this, suxamethonium-induced hyperkalemic cardiac arrest was still observed in a pregnant patient more than one month after recovering from Guillain-Barré syndrome [34]. This indicates an unpredictable serum-potassium response after successful treatment of a temporary risk factor.

### 5.5. Preexisting Hyperkalemia

Therapeutic doses of 1–1.5 mg/kg suxamethonium cause a serum-potassium increase of 0.5–1 mmol/L in healthy individuals [4]. The clinical implications of preexisting hyperkalemia prior to administration of suxamethonium are not well known but may depend on whether hyperkalemia developed acutely or gradually. In chronic hyperkalemia, physiologic adaptation may help to stabilize the myocardium and prevent ventricular arrhythmias rendering a slight acute rise in serum-potassium uneventful following administration of suxamethonium [35]. In contrast, further increase in serum-potassium on top of acute hyperkalemia is probably associated with substantial risk.

In time critical scenarios, suxamethonium is often administered before the serum-potassium level is known. Reports of giving suxamethonium to patients with serum-potassium levels of 5.6–7.2 mmol/L, unknown at the time of intubation, did not result in ECG changes [36] even in suxamethonium doses of 200 mg [37].

### 5.6. Rhabdomyosarcoma

Tumors of muscular origin may comprise large quantities of ACh receptors or ACh-like receptors. Case reports testify to lethal rises in serum-potassium levels following suxamethonium administration in patients with muscular tumors [38, 39]. Administration of suxamethonium to patients with sarcomas of unknown histological appearance or confirmed rhabdomyosarcomas should be performed cautiously.

### 5.7. Congenital Myopathy

Certain congenital myopathies are characterized by a muscle cell membrane vulnerability that may, in addition to the ACh receptor modulation described above, add an alternative pathophysiology to suxamethonium-associated hyperkalemia. In the vulnerable muscle cell membrane, extensive stress such as depolarization with suxamethonium may cause rhabdomyolysis and loss of intracellular contents including myoglobin, creatine kinase, and potassium to the extracellular space [5].

Cardiac arrest following suxamethonium has been recorded in children suffering from myopathies, occult at the time, such as Duchenne's, Becker, or unknown dystrophic muscular diseases [5, 40].

Children who have not reached their motoric milestones or have unexplained elevations in myoglobin or creatinine kinase may be suspected to have a congenital myopathy, and special considerations should be taken when planning the anesthetic strategy in this subgroup of patients.

## 6. Discussion

This short review describes the relationship between suxamethonium and hyperkalemia known for more than 50 years. Considering the vast number of annual anesthesia inductions using suxamethonium, the number of published case reports and reviews on the subject from the last two decades is very low. This indicates that the incidence of suxamethonium-induced clinically important hyperkalemia is extremely low, with increased physician awareness and the use of alternative neuromuscular blocking agents in high-risk patients.

The clinical appearance of hyperkalemia following suxamethonium administration correlates with its effects, rapid onset within a minute followed by a constant potassium release. Hyperkalemia may be suspected if bradycardia and widening of the QRS complexes appear on the ECG [15]. Treatment of hyperkalemia according to guidelines [41] should be initiated as soon as the diagnosis is confirmed. If cardiac arrest occurs, resuscitation may be highly resistant to adequate treatment [5] and extracorporal membrane oxygenation may be considered [42].

Due to better neurorehabilitation, longer ICU stays and the emergence of diseases may more often require reintubation, such as the coronavirus disease 2019 (COVID-19) disease [43], and the population at risk of hyperkalemia following suxamethonium administration may increase.

Also, during the current COVID-19 pandemic, shortages of anesthetic drugs may lead to local guidance on alternative drugs for ICU patients including the use of suxamethonium rather than rocuronium [22]. The number of fatalities which can be definitively linked to suxamethonium is few in the ICU, where possible causes of hemodynamic collapse during intubation are numerous. This may explain why some ICU physicians are unaware of suxamethonium-induced hyperkalemia [44]. The first case report of a Scandinavian COVID-19 patient experiencing lethal hyperkalemia following suxamethonium administration has been published [22]. This emphasizes the continued importance and actuality of the potentially deadly side effects of suxamethonium.

## 7. Conclusion

In the vast majority of patients, use of suxamethonium is safe. However, its use comes at a risk of excessive potassium release from the cytoplasm corresponding to the number of ACh receptors activated. In a selected subgroup of patients, this may be fatal. Patients at risk include upper and lower neuron injuries, major burns, trauma, immobility, prolonged critical illness, muscle tumors, and muscular dystrophy diseases. In patients with these conditions, an alternative neuromuscular blocking agent may be a more rational choice.

## Data Availability

The data generated or analyzed during this study are included within this article.

## Abbreviations

ACh: Acetylcholine
ICU: Intensive care unit
COVID-19: Coronavirus disease 2019.

## Additional Points

In high-risk individuals, administration of suxamethonium may lead to lethal hyperkalemia. Acetylcholine receptor modulation is the main cause of this phenomenon. Critical illness, extensive burns, and motor neuron lesions are major risk factors. It is essential to use an alternative muscle relaxant in high-risk patients.
